# Morphological analyses suggest a new taxonomic circumscription for *Hymenaea courbaril* L. (Leguminosae, Caesalpinioideae)

**DOI:** 10.3897/phytokeys.38.7408

**Published:** 2014-06-18

**Authors:** Isys Mascarenhas Souza, Ligia Silveira Funch, Luciano Paganucci de Queiroz

**Affiliations:** 1 Universidade Estadual de Feira de Santana, Programa de Pós-graduação em Botânica,Herbário, Km 03-BR 116, Campus. 44031-460, Feira de Santana, Bahia, Brasil

**Keywords:** Fabaceae, Detarieae, integrative taxonomy, nomenclatural changes

## Abstract

*Hymenaea* is a genus of the Resin-producing Clade of the tribe Detarieae (Leguminosae: Caesalpinioideae) with 14 species. *Hymenaea courbaril* is the most widespread species of the genus, ranging from southern Mexico to southeastern Brazil. As currently circumscribed, *Hymenaea courbaril* is a polytypic species with six varieties: var. *altissima*, var. *courbaril*, var. *longifolia*, var. *stilbocarpa*, var. *subsessilis*, and var. *villosa*. These varieties are distinguishable mostly by traits related to leaflet shape and indumentation, and calyx indumentation. We carried out morphometric analyses of 14 quantitative (continuous) leaf characters in order to assess the taxonomy of *Hymenaea courbaril* under the Unified Species Concept framework. Cluster analysis used the Unweighted Pair Group Method with Arithmetic Mean (UPGMA) based on Bray-Curtis dissimilarity matrices. Principal Component Analyses (PCA) were carried out based on the same morphometric matrix. Two sets of Analyses of Similarity and Non Parametric Multivariate Analysis of Variance were carried out to evaluate statistical support (1) for the major groups recovered using UPGMA and PCA, and (2) for the varieties. All analyses recovered three major groups coincident with (1) var. *altissima*, (2) var. *longifolia*, and (3) all other varieties. These results, together with geographical and habitat information, were taken as evidence of three separate metapopulation lineages recognized here as three distinct species. Nomenclatural adjustments, including reclassifying formerly misapplied types, are proposed.

## Introduction

*Hymenaea* L. is a genus of caesalpinioid legumes with 14 species (Lee and Langenheim 1975). The genus is distributed throughout tropical America, from Mexico to Paraguay, with one species in coastal East Africa (Mackinder 2005). *Hymenaea* is included in the Resin-producing Clade of the tribe Detarieae, and is most closely related to the genera *Guibourtia* Benn. and *Peltogyne* Vogel (Bruneau et al. 2001, 2008; Fougère-Danezan et al. 2007), all presenting similar leaf morphologies, with two asymmetrical pellucid-punctate leaflets. *Hymenaea* can be differentiated from these related genera by its rather larger and more massive bat-pollinated flowers with a robust hypanthium, and indehiscent, pulpy and woody pods with very large seeds.

The current taxonomy of the genus is largely based on Lee and Langenheim’s (1975) revision. In addition to recognizing the 14 currently accepted species, they reduced several species to varietal rank. These polytypic species were viewed by Lee and Langenheim (1975) as showing complex relationships with other species of *Hymenaea*. They hypothesized, for example, that *Hymenaea oblongifolia* Huber var. *oblongifolia* is more closely related to *Hymenaea aurea* Lee and Lang. and *Hymenaea eriogyne* Benth., while *Hymenaea oblongifolia* var. *davisii* (Sandwith) Lee and Lang. is probably more closely related to *Hymenaea parvifolia* Huber, *Hymenaea rubriflora* Ducke, and *Hymenaea reticulata* Ducke. According to their concepts, *Hymenaea oblongifolia* should be regarded as a polyphyletic species. Another example of a putative polyphyletic species, according to Lee and Langenheim’s (1975) conceptual framework, is *Hymenaea courbaril*, with var. *villosa* Lee and Andrade-Lima hypothesized as being more closely related to *Hymenaea martiana* Hayne, and var. *longifolia* (Benth.) Lee and Andrade-Lima to *Hymenaea velutina* Ducke and *Hymenaea stigonocarpa* Mart. ex Hayne.

*Hymenaea courbaril* is the most widely distributed species of the genus, almost matching the geographic range of *Hymenaea* in the New World. It also has the greatest economic importance in the genus, due to the high quality of its wood and its resin, the latter being used by native populations as incense, cement, in the manufacture of varnishes, and for medicinal purposes. Its nutritive fruits are sought after by mammals and birds (Rizzini 1971; Langenheim 1967; Lee and Langenheim 1975). *Hymenaea courbaril* is the most taxonomically complex species, with six varieties: var. *altissima* (Ducke) Lee and Lang., var. *courbaril*, var. *longifolia*, var. *stilbocarpa* (Hayne) Lee and Lang., var. *subsessilis* Ducke, and var. *villosa*. These varieties are differentiated by their leaflet sizes, shapes, and indumentation, calyx indumentation, petal shapes, ovary stipe sizes, and pod sizes and shapes. These variations in several diagnostic features make the boundaries of putatively related taxa rather imprecise. The widely circumscribed *Hymenaea courbaril*, as defined by Lee and Langenheim (1975), is hereafter referred to as the *Hymenaea courbaril* complex.

The species and varietal limits of *Hymenaea courbaril*, *Hymenaea stigonocarpa*, and *Hymenaea martiana* were investigated by Pestana (2010). This author did not employ objective analytical methods and used the same classical taxonomic approach as Lee and Langenheim (1975), thus coming to similar conclusions as the latter authors in keeping *Hymenaea courbaril* as a polytypic species with six varieties.

Delineating precise species boundaries is a key task in plant taxonomy. This process has direct impacts on society, as there is a growing demand for credible taxonomic information that allows us to conserve, manage, and understand natural biodiversity (Wheeler et al. 2004). However, questions of species recognition can be affected by several theoretical, methodological, and practical issues. De Queiroz (2005, 2007) defined species as separately evolving metapopulation lineages (the Unified Species Concept–USC), and proposed that all other previously considered properties of species should be reinterpreted as contingent rather than critical. These additional contingent properties, such as phenetic distinctiveness, reciprocal monophyly, genetic coalescence, or ecological distinctiveness, are acquired during speciation and should be considered as different lines of evidence relevant to assessing lineage separation. The USC, by treating species conceptualization and species delimitation as clearly separate issues, allows the use of properties formerly treated as secondary criteria in species delimitations. More importantly, it allows for currently accepted species limits to be considered as hypotheses to be tested using the presence of any one of those secondary properties as evidence for the existence of a species.

Analyses of morphometric data can be useful in objectively demonstrating species limits, especially when combined with molecular markers (Andrés-Sánchez et al. 2009; Newmaster and Ragupathy 2009). Additionally, the use of morphological information represents the fastest and least expensive manner of assessing taxonomic complexes–and has been used to solve problems of species limits in many different plant groups, especially when molecular data was not easily available (Handerson 2006; Estrella et al. 2009; Pedersen 2010; Ceolin and Miotto 2012; Rahman and Rahman 2012; Castello and Galetto 2013; Scrivanti et al. 2013), as was the case of the group studied here.

The problem of defining species limits in the polytypic *Hymenaea courbaril* complex is revisited here under the USC conceptual framework by exploring morphometric, geographical, and ecological patterns as lines of evidence for the existence of separate metapopulation lineages. Specifically, we sought to test Lee and Langenheim’s (1975) hypothesis that *Hymenaea courbaril* should be treated as a polytypic species with six varieties.

## Materials and methods

A total of 96 specimens of the *Hymenaea courbaril* complex were examined in this study (vouchers listed in Appendix 1). All analyzed materials were sheets from the following herbaria: B, CEN, CEPEC, HRB, HUEFS, IBGE, IPA, LAGU, M, MBM, NY, RB, SP, SPF, U, UB, and UC. We selected specimens that displayed branch ends with fully-developed (mature) leaves. This criterion avoided considering young leaves from the tips of the branches or leaves at the bases of the branches that are often much larger. Each specimen analyzed was considered an individual, and identifications strictly followed Lee and Langenheim (1975), although these names were only used as nomenclatural references in this study.

**Figure 1.**
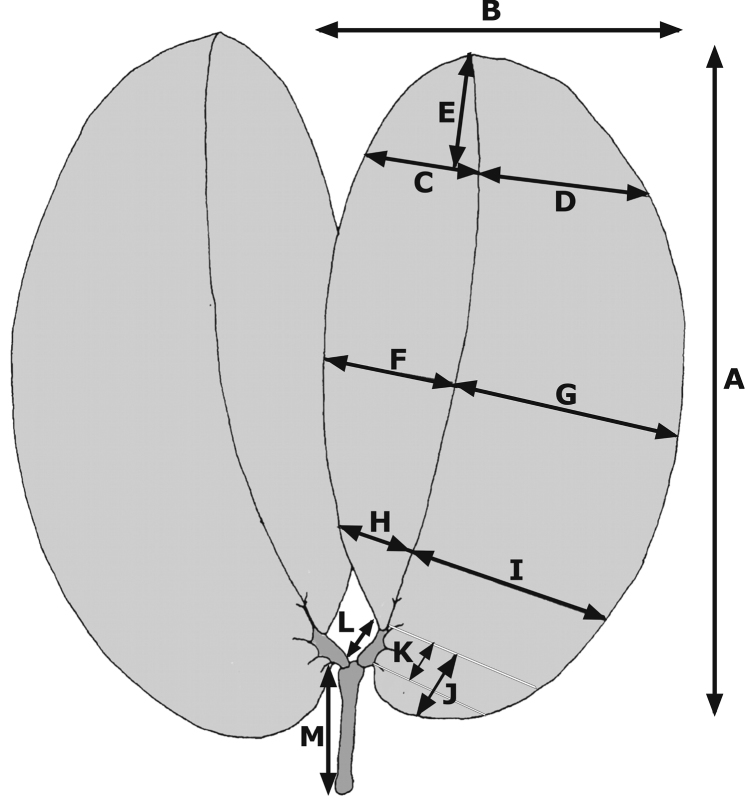
Quantitative leaf characters of specimens of the *Hymenaea courbaril* complex. Letters refer to the measurements described in Table 1.

The leaves in the group studied here are bifoliolate; the leaflets range from oblong to ovate or obovate, with rounded, acute or obtuse apices; the main vein is displaced towards the inner margin resulting in an asymmetrical base, the outer portion being wider than the inner and extending beyond the attachment to the petiolule (Figure 1). Fourteen quantitative (continuous) characters were examined (Table 1; Figure 1). Only leaf traits were measured and quantified, as most herbarium sheets lacked flowers and/or fruits. In any case, flower morphology is much conserved in the species studied and the herbarium material examined usually contained only incomplete or damaged flowers. Measurements were taken of two fully developed leaves per dried herbarium sheet, using a graduated ruler (precision 1 mm).

**Table 1.** List of the quantitative leaf characters in specimens of the *Hymenaea courbaril* complex. Letters in the second column refer to measurements depicted in Figure 14.

Leaf characters (cm)	Measurements in Figure 1
1 - Total length of the leaflet	A
2 - Leaflet length /width ratio	A / B
3 - Length of the distal sixth	E
4 - Inner width in distal third	C
5 - Outer width in distal third	D
6 - Inner width in middle third	F
7 - Outer width in middle third	G
8 - Inner width in basal third	H
9 - Outer width in basal third	I
10 - Main vein displacement	(G–F) / (I–H)
11 - Base extension (length of the base from the inner attachment of the petiolule)	J
12 - Petiole length	M
13 - Petiolule length	L
14 - Distance between inner and outer attachment of the petiolule	K

All multivariate analyses were carried out using Past software (Hammer et al. 2001). Cluster analyses used the Unweighted Pair Group Method with Arithmetic Mean (UPGMA) based on Bray-Curtis dissimilarity matrices. Principal Component Analyses (PCA) were carried out based on the same morphometric matrix. Two sets of Analyses of Similarity (ANOSIM; Warwick, Clarke and Suharsono 1990) and Non Parametric Multivariate Analysis of Variance (NPMANOVA; Anderson 2001) were carried out to evaluate statistical support for: (1) the major groups recovered in the UPGMA and PCA; and (2) the varieties as defined by Lee and Langenheim (1975). *Hymenaea courbaril* var. *villosa* was not included in the second analysis because it is known from only two specimens (Lee and Langenheim 1975) and only one was located during this study. Both ANOSIM and NPMANOVA used Bonferroni corrections, 10,000 permutations, and Bray-Curtis distances. As *Hymenaea courbaril* var. *villosa* was represented by only a single specimen, we carried out a second set of UPGMA, PCA, and similarity analyses for the major groups without including this variety in order to test its influence on the results.

Distribution maps of the specimens studied were prepared using DIVA-GIS software (Hijmanns et al. 2005), based on the geographic coordinates recorded on the herbarium sheet labels. For material lacking original coordinates, a central coordinate for the municipality was used as provided by the Species Link website (available at http://specieslink.org.br ).

Species limits were tested following the USC framework (de Queiroz 2005, 2007). Species ranks were ascribed to groups that showed morphological and habitat distinctiveness and geographical consistency. We considered as morphologically distinct groups those that were recovered in UPGMA and PCA and that exhibited statistical significance in both ANOSIM and NPMANOVA tests. Habitat distinctiveness was assessed from the vegetation type where the taxon occurs, following the UNESCO (1973) classification. Species diagnoses were prepared based on vegetative and reproductive characters.

## Results and discussion

**Figure 2.**
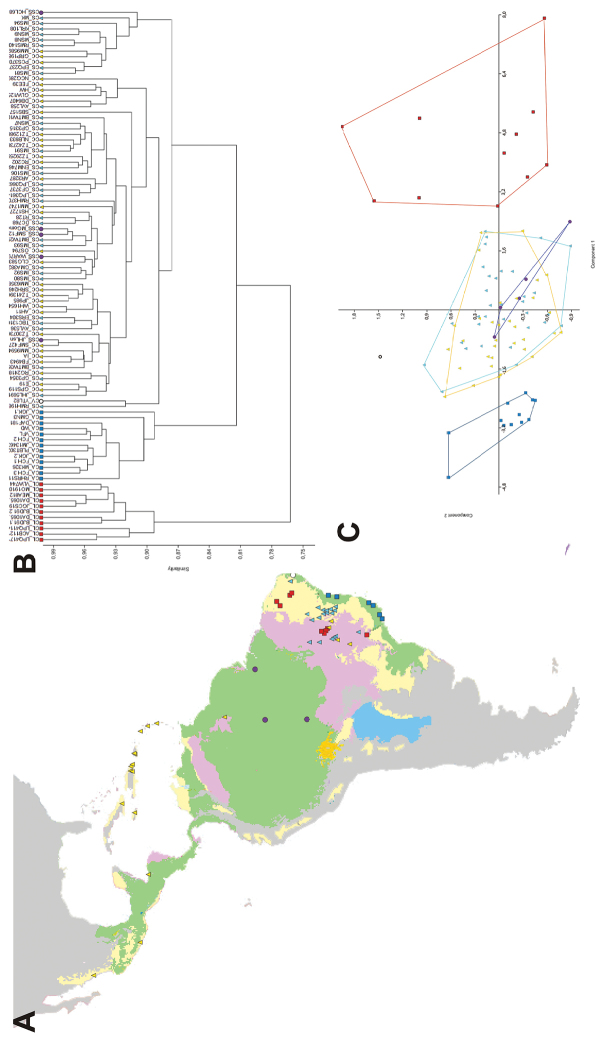
**A** Distribution of the *Hymenaea courbaril* complex in the Neotropics. The major biomes where this complex occurs are shown in color: wet forests (green), seasonally dry forests and woodlands (yellow), and savannas (purple). The varieties of *Hymenaea courbaril* recognized by Lee and Langenheim (1975) are represented by different symbols: var. *altissima* (blue squares), var. *coubaril* (yellow triangles), var. *longifolia* (red squares), var. *stilbocarpa* (blue triangles), var. *subsessilis* (purple circles), and var. *villosa* (white circles) **B** UPGMA analysis of the *Hymenaea courbaril* complex based on 14 quantitative leaf measures (see Figure 1 and Table 1 for measurement details and Bray-Curtis distances). Varieties are represented by the same symbols used for the map **C** Scatter diagram showing the first two axes of the PCA using the same data matrix as the UPGMA analysis. Ellipses represent the varieties as recognized by Lee and Langenheim (1975), and they are represented by the same symbols used for the map.

**Figure 3.**
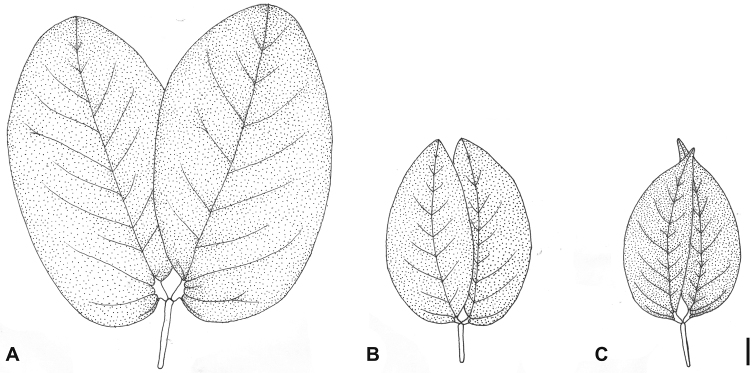
Leaf morphology of the species recognized in the *Hymenaea courbaril* complex: **A** *Hymenaea longifolia* **B** *Hymenaea courbaril* **C** *Hymenaea altissima*. Scale bar = 1 cm.

Both UPGMA and PCA recovered three major groups (Figure 2): Group 1 included all specimens of var. *altissima*; Group 2 all specimens of varieties *courbaril*, *stilbocarpa*, *subsessilis*, and the single specimen of *villosa*; and Group 3 all specimens of var. *longifolia*. Within Group 2, individuals of the different varieties did not cluster together and appeared intermixed in UPGMA, or formed highly overlapping groups in two first axes of PCA. The first PCA axis accumulated 88.3% of the total variance, with the two first axes summing 93% of the observed variation. Leaflet length was the trait that explained most of the variation found in first axis, and the three major groups were sorted mostly by leaflet size. These results indicate that Group 3 includes specimens with largest leaflets, and Group 1 the smallest leaflets (Figure 3).

The results of the UPGMA and PCA were consistent with both ANOSIM and NPMANOVA. Comparisons of the varieties of *Hymenaea courbaril* showed significant differences (p < 0.05) between the varieties *altissima* and *longifolia* and all other varieties, but no significant differences between the varieties *courbaril*, *silbocarpa*, and *subsessilis* that clustered in Group 2 (Table 2). The same analyses comparing the three groups recovered in UPGMA and PCA found significant morphological differences between them (Table 3).

**Table 2.** Results of ANOSIM and NPMANOVA testing the consistence of the varieties of *Hymenaea courbaril*, using 10,000 permutations and Bray-Curtis distances. ANOSIM R-values are given above the diagonal and NPMANOVA F-values are given below the diagonal. Numbers between brackets are Bonferroni-corrected p-values. Significant differences are indicated by shadowed cells.

	stilbocarpa	courbaril	subsessilis	altissima	longifolia
stilbocarpa		0.09933 [0.0195]	-0.1343 [1]	0.9325 [0.0015]	0.8858 [0.0015]
courbaril	5.313 [0.0855]		0.09375 [1]	0.8524 [0.0015]	0.9471 [0.0015]
subsessilis	0.8397 [1]	3.296 [0.489]		0.9972 [0.0045]	0.9329 [0.0075]
altissima	79.79 [0.0015]	57.97 [0.0015]	49.86 [0.003]		1 [0.0015]
longifolia	67.13 [0.0015]	94.08 [0.0015]	30.27 [0.006]	235.5 [0.0015]	

**Table 3.** ANOSIM and NPMANOVA (10,000 permutations and Bray-Curtis distances) for the groups recovered in UPGMA and PCA analyses of 78 individuals of *Hymenaea courbaril* complex. Group 1 included all specimens of var. *altissima*, Group 2 all specimens of the varieties *courbaril*, *stilbocarpa*, *subsessilis*, and *villosa*, and Group 3 all specimens of var. *longifolia*. Values before the slash resulted from the analyses including var. *villosa* and after one excluding it. ANOSIM R-values are given above the diagonal and NPMANOVA F-values are given below the diagonal. Numbers in brackets are Bonferroni-corrected p-values. Significant differences are indicated by shadowed cells.

	Group 1	Group 2	Group 3
Group 1		0.8762 / 0.8771 [0.0003 / 0.0003]	1 / 1 [0.0003 / 0.0003]
Group 2	73.65 / 74.35 [0.0003 / 0.0003]		0.9045 / 0.9055 [0.0003 / 0.0003]
Group 3	235.5 / 235.5 [0.0003 / 0.0003]	84.47 / 85.58 [0.0003 / 0.0003]	

The complex morphological variations found in Group 2 appear to reflect its wide geographic range and large genetic variability (Ramos et al. 2009). Phylogeographic studies in part of the geographic range of *Hymenaea courbaril* var. *stilbocarpa* clearly demonstrated the wide genetic base and geographic structure of this genetic variation (Ramos et al. 2009). The morphological distinctions among the varieties clustered in Group 2 are made by rather continuous characters, such as the color of the calyx lobes (ochraceous to golden or rusty brown), leaflet shape (broadly to narrowly falcate), and the shapes of the outer sides of the leaflet base (straight to nearly straight or rounded). The var. *subsessilis* presents a short ovary stipe (c. 2 mm long) that grades to a medium-sized stipe in var. *stilbocarpa* (3–4 mm) and then large in var. *courbaril* (4–6 mm).

Leaflet indument distinguishes var. *villosa* from the remaining varieties of Group 2 (Lee and Andrade-Lima 1974, 1975). The ovary is also described as slightly pilose on one side, a condition not observed in other varieties of *Hymenaea courbaril*. The exclusion of var. *villosa* from the similarity analyses did not alter the results obtained with its inclusion (Table 3). The var. *villosa* is known from only two specimens from the eastern coastal areas of the states of Paraíba and Pernambuco (northeastern Brazil) where the range of var. *stilbocarpa* overlaps with that of *Hymenaea martiana*. This species (*Hymenaea martiana*) has tomentose leaflets and an ovary with a tuft of trichomes near its base. The rarity of *Hymenaea courbaril* var. *villosa*, as well as the transitional nature of the leaflets and ovary indumentation in relation to *Hymenaea martiana* and the other varieties of the *Hymenaea courbaril* complex clustered in Group 2, all suggest that var. *villosa* could represent a hybrid between *Hymenaea martiana* and *Hymenaea courbaril*.

The habitats of the taxa of Group 2 include mostly tropical ombrophilous alluvial (gallery) forests in areas subject to seasonally dry climates from Mexico and the Caribbean islands to central Brazil, but not the Amazonian region (var. *courbaril* and *stilbocarpa*), tropical ombrophilous lowland forests in central and eastern Amazon basin (var. *subsessilis*), or tropical ombrophilous submontane forests in the northeastern Brazilian state of Paraíba (var. *villosa*) (Figure 2).

The var. *altissima* (Group 1) constitutes a morphologically, geographically and ecologically consistent taxon. It is distinguished from the other varieties of *Hymenaea courbaril* complex by having smaller, falcate and acuminate leaflets, not exceeding 6.5 cm long on the flowering branches (Table 4). The leaflets of this taxon have a thinner texture than the remaining varieties, allowing its tertiary venation to appear as raised and reticulate veins. The flowers of var. *altissima* are also the smallest within the *Hymenaea courbaril* complex, measuring less than 15 mm long (Lee and Langenheim 1975, Pestana 2010). This variety is distributed in tropical ombrophilous lowland and submontane forests of the Atlantic Forest phytogeographical domain along the eastern coast of Brazil (Figure 2). It was cited for the southeastern Brazilian states of São Paulo and Rio de Janeiro (Mattos 1968, Lee and Langenheim 1975, Rizzini 1978, Pestana 2010), but we found specimens occurring northwards into Espírito Santo and Bahia states. It was originally described as *Hymenaea altissima* Ducke (Ducke 1935). Lee and Langenheim (1974: 448) considered that the foliar characters and relative flower size “do not appear sufficiently significant to warrant specific status for this taxon” and considered it as a variety of *Hymenaea courbaril*.

**Table 4.** Morphological comparison between *Hymenaea courbaril*, *Hymenaea altissima*, and *Hymenaea longifolia*.

	*Hymenaea courbaril*	*Hymenaea altissima*	*Hymenaea longifolia*
Leaflet outline	Elliptic or ovate, not or slightly falcate	Falcately elliptic	Oblong or narrowly elliptic, not falcate
Inner margin of the leaflet	Straight or slightly concave	Strongly concave	Straight
Outer base of the leaflet	Slightly rounded	Truncate	Rounded
Apex	Acute, rarely obtuse	Abruptly acuminate	Obtuse, rarely rounded
Petiole length (cm)	1.1–1.7 (– 2)	1–1.3 (– 1.7)	2.6–3
Petiolule length (mm)	4–5	3–4 (– 5)	7–8
Leaflet size (cm)	6–9 (– 10.2) × 2.8–4 (– 5.1)	4–5.7 (– 6.4) × 2.0–2.5	10.1–12.5 (–15.4) × 4.5–6.5 (– 6.9)
Flower length (cm)	2.2–3.1	2–2.7	2.6–3.8
Fruit shape	Cylindrical, rounded in cross section	Cylindrical, rounded in cross section	Oblongoid, compressed in cross section
Seeds per fruit	Mostly > 4	Mostly 1–2	Mostly > 4

Var. *longifolia* (Group 3) comprises the morphs with largest leaflets. This variety also differs from the remaining groups by having oblong leaflets with an obtuse apex, clawed petals, and fruits compressed and enlarged toward the apex (Table 4). It occupies a very particular habitat in submontane tropical drought-deciduous thorny forests in the Caatinga and Cerrado phytogeographical domains that extend from western Bahia and Piauí states to the Araripe mountain range in the border area between the states of Ceará and Pernambuco (Figure 2). It was first proposed as a variety of *Hymenaea splendida* Vogel. Lee and Langenheim (1974, 1975) considered *Hymenaea splendida* var. *splendida* to be synonymous with *Hymenaea courbaril* var. *stilbocarpa* and segregated *Hymenaea splendida* var. *longifolia* as another variety of *Hymenaea courbaril*.

In addition to morphological distinctions, ecological and geographical information can be used for interpreting the three groups recovered by UPGMA and PCA as separately evolving lineages that would be considered different species under the USC framework (see Taxonomy section). With respect to the variation in Group 2, studies of wider samplings, including especially the collections made after Lee and Langenheim’s (1975) work, shows that the diagnostic features used for defining the varieties included in Group 2 have more complex variations. Indumented leaflets, for example, the diagnostic character for var. *villosa*, can be found to varying degrees in var. *stilbocarpa*. A short stipe was used as a diagnostic feature of var. *subsessilis*, but this trait is variable and grades into var. *courbaril*. Finally, the distinctions between vars. *courbaril* and *stilbocarpa* rely on the types and colors of the calyx lobe indumentation, traits that tend to change during the duration of the flower. Thus, a more parsimonious way to treat the taxonomy of Group 2 is to consider it a variable species, without recognizing varieties.

## Taxonomic treatment

### Hymenaea courbaril

L., Sp. Pl. 1192. 1753. Lectotype. “Ceratia diphyllos Antegoana, Ricini majoris, fructu osseo, siliqua grandi incluso” in Plukenet, Phytographia, t. 82, f. 3, 1691 (designated by Lee and Langenheim in Univ. Calif. Publ. Bot. 69: 81. 1975).

Hymenaea resinifera Salisb., Prodr. 327. 1796, *nom. nud*.
? Hymenaea animifera Stokes, Bot. Mat. Med. 2: 449. 1812, *fide* Lee and Langenheim in Univ. Calif. Publ. Bot. 69: 81. 1975.
Hymenaea candolleana Kunth [spelled ‘*candolliana’*], Nov. Gen. and Sp. 6: 323, pl 556. 1824. Type: México, Acapulco, *Bonpland 3875* (holotype P!).
Hymenaea confertifolia Hayne, Getreue Darstell. Gew., 11, pl. 9. 1830. Lectotype (designated here): *Sellow* s.n., s.d. (lectotype W 48857!).
Hymenaea stilbocarpa Hayne, Getreue Darstell. Gew., 11, pl. 11. 1830. *Hymenaea courbaril* var. *stilbocarpa* (Hayne) Lee and Lang., J. Arnold Arbor. 55: 449. 1974, syn. nov. Lectotype (designated here): “S. Pauli, sylv. ad Faz. de S. Anna”, *Martius* (lectotype M! [barcode n° M-0215314], photo HUEFS!, isolectotype M! [barcode n° M-0215313], photo HUEFS!).
Hymenaea retusa Willd. ex Hayne, Darst. Beschreib. Arzneigew. 11: pl. 12. 1830. Type: México, Acapulco, *Humboldt* (holotype B!), *nom. illegit*.
Hymenaea splendida Vogel, Linnaea 11: 409. 1837. Type: *Sellow 1025* (holotype B†).
Hymenaea courbaril var. *obtusifolia* Ducke, Arch. Jard. Bot. Rio de Janeiro, 4: 47. 1925. Type: Brazil, Pará, Belém (Ilha do Marajó?), *Ducke RB 16906* (holotype RB!, isotypes A!, MG!, P!, U!, US!).
Hymenaea courbaril var. *subsessilis* Ducke, Arch. Jard. Bot. Rio de Janeiro 4: 265. 1925, syn. nov. Neotype (designated by Lee and Langenheim in Univ. Calif. Publ. Bot. 69: 89. 1975): Brasil, Amazonas, Flores, *Ducke MG 11167* (neotype MG!).
Hymenaea multiflora Kleinhoonte, Recueil Trav. Bot. Neerl. 22: 405. 1925. Type: Suriname, Boschreserve, Kaboeri, Coll. by the Forestry Bureau Herb. 4943 (holotype U!).
Inga megacarpa M.E. Jones, Contr. West Bot. 15: 140. 1929. Type: México, Nayarit, *M.E. Jones 23012* (holotype RSA!, isotypes F!, GH!, NY!).
Hymenaea courbaril var. *villosa* Y.T. Lee and Andrade-Lima, J. Arnold Arbor. 55: 446. 1974, syn. nov. Type: Brasil, Paraíba, Paquevira de Natuba, *Lee and Andrade-Lima 83* (holotype UC!).

#### Description.

Large trees, to 30 m tall. Petiole 1.1–1.7 (–2) cm long; petiolule 4–5 mm long; leaflets 6–9 (–10.2) × 2.8–4 (–5.1) cm, elliptic, rarely ovate, straight or slightly falcate due to central vein with an angle c. 10°, apex acute or obtuse, rarely acuminate, base acute along the inner margin and rounded along the outer margin, distance from the inner margin to central vein 7–10 mm in the basal region. Flower buds 2.1–2.5 cm long; flower 2.2–3.1 cm long; hypanthium 7–15 mm long; petals 1.1–2.0 cm long. Fruit cylindrical, of uniform width, apex mostly rounded and apiculate.

*Hymenaea courbaril* is defined here more narrowly than the circumscription adopted by Lee and Langenheim (1975), as we are proposing the exclusion of the varieties *altissima* and *longifolia* and their recognition as distinct species. In this narrower sense, *Hymenaea courbaril* is characterized by leaflets with slightly convex inner margins and midrib slightly arched, resulting in an elliptic or ovate outline, not or only slightly falcate, apex mostly acute or obtuse (Figure 3). It presents flowers larger than the other species of the *Hymenaea courbaril* complex, and cylindrical fruits mostly with more than five seeds (Table 4). It has a wide geographical range, mostly in different nuclei of Seasonally Dry Forests in Mexico, Central America, Caribbean, northern South America, Bolivia, and Brazil (from the Amazon region to Paraná State in the south).

Nomenclatural problems with several names associated with *Hymenaea courbaril* were not amended by Lee and Langenheim (1975) when they undertook their taxonomic revision of the genus. No formal type for *Hymenaea stilbocarpa* was cited by Lee and Langenheim (1975), who stated that no specimen was cited in the original description of Hayne (1830). However, Hayne (1830) based *Hymenaea stilbocarpa* on material collected by Martius, citing “Wäscht in Brasilien in Wäldern der Provinzen S. Paulo, Minas Geraes und Bahia (Martius)”. We found a specimen in the M herbarium collected by Martius in the Brazilian state of São Paulo (Santana farm) with an attached label indicating that this plant was distributed over the “Prov. Rio de Jº., S. Paulo, Minas Geraes, Bahia”, which probably served as the original material for Hayne’s description of *Hymenaea stilbocarpa*. Thus, we are lectotypifying this species with Martius’ specimen held in M under the barcode number M-0215314.

*Hymenaea confertifolia* Hayne was based on material collected by Sellow and Olfers in Brazil (“Wächst in Brasilien (Olfers u. Sellow)”; Hayne 1830: table 9). Lee and Langenheim (1975: 88) stated that this name was based on *Sellow 1025* and that the “holotype” in the B herbarium was destroyed. However, as Hayne (1830) did not refer to one particular specimen, all materials collected by Sellow or Olfers that can be linked with *Hymenaea confertifolia* should be considered syntypes. No such specimens can be found in the B herbarium, and were probably destroyed. A duplicate from B collected by Sellow was found in W annotated as *Hymenaea confertifolia*, and is designated here as the lectotype of this name.

*Hymenaea retusa* Willd. ex Hayne was published as a homotypic synonym of *Hymenaea candolleana* (Hayne 1830). It is thus illegitimate under the Article 52 of the International Code of Botanical Nomenclature (McNeill et al. 2011).

*Hymenaea courbaril* var. *obtusifolia* Ducke was published based on a tree cultivated in the Pará Botanical Garden (now Museu Paraense Emílio Goeldi, Belém, state of Pará, Brazil). Lee and Langenheim (1975) misinterpreted this as evidence that the holotype was the specimen in the herbarium of this museum (MG). However, Ducke (1925: 47) explicitly stated that “specimina florifera and fructus in herb. Jard. Bot. Rio n. 16.906”, and thus the RB material should be considered as the holotype of this name.

Ducke (1925: 265) did not cite any specimen when describing *Hymenaea courbaril* var. *subsessilis* Ducke. Lee and Langenheim (1975) did not explicitly designate a type for this variety, but chose a “representative specimen” collected “in the same general area believed to be the type locality”. We are accepting it as an inferential typification, and the status of this material should be a neotype since no other specimen was refereed in the protologue.

### Hymenaea altissima

Ducke, Anais Acad. Brasil. Ci. 7: 207. 1935. Lectotype (designated by Egler in Bol. Mus. Emílio Goeldi. Nov. Ser. Botânica. 18: 51. 1963): Brazil, Rio de Janeiro, Avelar, Faz. Pau Grande, Posse, G. M. Nunes 3 (lectotype RB!, isolectotypes MG!, R!).

Hymenaea courbaril var. *altissima* (Ducke) Lee and Lang., J. Arnold Arbor. 55: 448. 1974.

#### Description. 

Large trees, up to 38 m tall. Petiole 1–1.3 (–1.7) cm long; petiolule 3–4 (–5) mm long; leaflets 4–5.7 (–6.4) × 2–2.5 cm, oval, narrow-elliptic or elliptic, strongly falcate, due to central vein with an angle c. 35°, apex acuminate, rarely acute, base acute along the inner margin and truncate along the outer margin, distance from the inner margin to central vein 4–5 mm in the basal region. Flower buds 1.5–2.5 cm long; flower 2–2.7 cm long; hypanthium 7–12 mm long; petals 1.2–1.6 cm long. Fruit cylindrical, of uniform width, apex mostly rounded and apiculate.

*Hymenaea altissima* shows the smallest leaflets and flowers in the species group related to *Hymenaea courbaril* (Table 4). Additionally, its leaflets are strongly falcate due to the strongly curved and displaced midvein, with an abruptly acuminate apex. The base of the leaflet is acute along the inner margin and truncate along the outer margin (Figure 3). The fruits are mostly cylindrical, as in *Hymenaea courbaril*, but usually shorter than those of this species (4–5 vs. 8–20 cm long) because they have only one or two seeds, while in *Hymenaea courbaril* they usually have six or more seeds. This species is restricted to the coastal rain forests of eastern Brazil, from southern Bahia State to São Paulo and Paraná states.

This species was described by Ducke (1935) as *Hymenaea altissima*. Lee and Langenheim (1974) treated it as a variety of *Hymenaea courbaril*. Later, these latter authors highlighted the differences between this taxon and the other varieties as having smaller and falcate leaflets and smaller flowers (Lee and Langenheim 1975: 86), but kept it as a variety of *Hymenaea courbaril*, a position also adopted by Pestana (2010). Our results indicated that the leaf traits of *Hymenaea altissima* are clearly distinct of those of *Hymenaea courbaril* and *Hymenaea longifolia*. These results, together with the distinctive flower and fruit traits and the coherent distribution and habitat data, all give support to the original view of Ducke (1935) in considering it a different species.

Lee and Langenheim (1975: 84) stated that the lectotype of *Hymenaea altissima* was designated by Egler (1963) as the specimen “*A. Ducke RB 30306*”. However, Egler (1963: 51) simply indicated that the type should be “Type: RB 23.306”. We could not track any specimens of *Hymenaea* in the RB herbarium with the numbers RB 30306 or RB 23306. We encountered, however, a specimen annotated by Ducke as *Hymenaea altissima* that was collected in the state of “Rio de Janeiro, Avelar” by G. M. Nunes in 1925 and could be linked to the protologue of *Hymenaea altissima* (Ducke 1935). This specimen is numbered RB 20306, and we are assuming that both Egler (1963) and Lee and Langenheim (1975) erroneously noted the RB number when referring to the lectotype of *Hymenaea altissima*.

### Hymenaea longifolia

(Benth.) I.M. Souza, Funch & L.P. Queiroz comb. et stat. nov.

urn:lsid:ipni.org:names:77140226-1

Hymenaea splendida var. *longifolia* Benth., Fl. Bras. (Martius) 15(2): 236. 1870. *Hymenaea courbaril* var. *longifolia* (Benth.) Y.T.Lee and Andrade-Lima, J. Arnold Arbor. 55: 448. 1974. Lectotype (designated here): Brasil, Bahia, Villa de Barra, *Blanchet 3135* (lectotype: R!, isolectotypes K!, P!).

#### Description. 

Medium-sized trees c. 5–12 (–18) m tall. Petiole 2.6–3 cm long; petiolule 7–8 mm long; leaflets 10.1–12.5 (–15.4) × 4.5–6.5 (–6.9) cm, elliptic, narrow-elliptic, or oblong, not falcate, due to central vein with an angle c. 24°, apex obtuse or acute, base acute or rounded along the inner margin and rounded or slightly rounded along the outer margin, distance from the inner margin to central vein 10–11 mm in the basal region. Flower buds 2.2–3.3 cm long; flower 2.6–3.8 cm long; hypanthium 9–14 mm long; petals 1.3–2.1 cm long. Fruit oblongoid, compressed, wider at distal region, apex slightly truncate and apiculate.

*Hymenaea longifolia*, as circumscribed here, is characterized by large leaflets, both longer and wider than those of the related species *Hymenaea courbaril* and *Hymenaea altissima*, with longer petioles and petiolules (Table 4). The leaflets are elliptic or oblong with obtuse (rarely acute) apices and rounded bases along the outer margin (Figure 3). This species occurs in Seasonally Dry Forests within the Caatinga and Cerrado phytogeographical domains in northeastern Brazil, from Ceará State to the northern portion of Bahia State.

It was first described by Bentham (1870) as *Hymenaea splendida* var. *longifolia*, and considered related to *Hymenaea courbaril* and *Hymenaea stilbocarpa* because of the glabrous leaflets, but differing from them by having larger leaflets. Lee and Langenheim (1975) treated all these taxa under a more widely circumscribed *Hymenaea courbaril*, a position not supported by the results presented here. Lee and Langenheim (1975: 86) misinterpreted the specimen *Blanchet 3135* (R) as the holotype of *Hymenaea splendida* var. *longifolia*. However, when describing this variety, Bentham (1870: 236) cited two syntypes, *Blanchet 3135* (“ad Villa da Barra prov. Bahia”) and *Gardner 1938* (“Serra da Araripé, prov. Ceara”). Thus, the material cited by Lee and Langenheim (1975) as the holotype should be considered as a lectotype.

## Supplementary Material

XML Treatment for Hymenaea courbaril

XML Treatment for Hymenaea altissima

XML Treatment for Hymenaea longifolia

## Appendix 1

Materials examined for the morphometric study of the *Hymenaea courbaril* complex. Nomenclature follows Lee and Langenheim (1975).

***Hymenaea courbaril* var. *altissima–*BRAZIL, Bahia**, Ilhéus, *R.H.R.Sambuichi 1169* (CEPEC). **Esprírito Santo**, Linhares, *D.A.Folli 181* (RB). **Rio de Janeiro**, Nova Iguaçu, *W.Dias s/n* (HUEFS); Resende, *J.G.Kuhlmann 179* (RB); Rio de Janeiro, *J.G.Kuhlmann s/n* (RB). **São Paulo**, Moji das Cruzes, *P.L.B.Tomasulo 307* (SP); São Paulo, *F.C.Hoehne s/n* (SP, SPF); *J.Mattos 13463* (SP); *M. Kirizawa 326* (SP); *V.F.Lima s/n* (SP).

***Hymenaea courbaril* var. *courbaril–*BRAZIL, Bahia**, Baianópolis, *S.B. da Silva 157* (HRB). Bom Jesus da Lapa, *S.M. de Faria 427* (RB). **Goiás**, Niquelândia, *G.P.Silva 1192* (CEN); Santa Bárbara de Goiás, *A.Raw 3287* (UB). **COLOMBIA**, Santa Maria, *Espina 19* (UC). **CUBA, Santiago de Cuba**, Mella, *I.Arias s/n* (B). **EL SALVADOR**, s/l, *R.Cruz 202* (LAGU); *P.C.Standley 3705* (UC). **HONDURAS, Morazan**, Jicarito, *G.L.Webster 12529* (F). **MEXICO**, Acapulco, *W. Humboldt s/n* (B). **PANAMA**, Barro Colorado, *N.C.Garwood 2891A* (F); *O.Shattuck 794* (UC). **SURINAME**, Boschreserve, *F.Bureau 4943* (U).

***Hymenaea courbaril* var. *longifolia–*BRAZIL, Bahia**, Barreiras, *L.P. de Queiroz 4114* (HUEFS); Formosa do Rio Preto, *D.Alvarenga 1065* (RB); *B.J.Dias 91* (IBGE, SP); *L.P. de Queiroz 4171* (HUEFS). **Ceará**, Brejo Santo, *J.G.Carvalho-Sobrinho 1925* (HUEFS); Missão Velha, *Academia Brasileira de Ciências 1121* (IPA). **Piauí**, Brasileira, *M.E.Alencar 812* (HUEFS); Campo Maior, *M.Oliveira 1910* (IPA). **São Paulo**, Bauru, *V. de L. Weiser 744* (RB).

***Hymenaea courbaril* var. *stilbocarpa–*BRAZIL, Bahia**, Caetité, *L.P. de Queiroz 3614* (HUEFS); Gentio do Ouro, *K.R.B.Leite 108* (HUEFS); *E.R. de Souza 304* (HUEFS); *R.Tourinho 28* (HUEFS); Jaguaripe, *E.N. de Matos 746* (HUEFS); Livramento do Brumado, *R.M.Harley 19889* (IPA); *L.P. de Queiroz 3663* (HUEFS); Miguel Calmon, *E.P.Queiroz 2372* (MBM); Mucugê, *R.M.Harley 3702* (HUEFS); Palmeiras, *M. de S. Nunes 7, 8, 9* (HUEFS); *I.M.Souza 80, 81, 91, 92, 93, 94, 106* (HUEFS); Rui Barbosa, *D.Cardoso 768* (HUEFS); Vitória da Conquista, *J.H.Langenheim 5641* (IPA). **Goiás**, Alto Paraíso de Goiás, *T.B.C. 1310* (HUEFS); Colinas do Sul, *B.M.T.Walter 1038* (HUEFS); Minaçu, *B.M.T.Walter 3570* (HUEFS); Niquelândia, *B.M.T.Walter 2515* (HUEFS). **Minas Gerais**, Araguari, *G.M.Araújo 382* (NY). **Paraíba**, São João do Cariri, *A.V.Lacerda 258, 536* (HUEFS). **Pernambuco**, Chapada do Araripe, *G.Fotius 3737* (HUEFS). **Piauí**, Caracol, *R.M.Santos 1400* (HUEFS). **São Paulo**, São Paulo, *M.Koscinski s/n* (IPA). **Tocantins**, Colméia, *G.Pedralli 3315* (HUEFS); Santa Rosa do Tocantins, *G.Pedralli 3354* (HUEFS).

***Hymenaea courbaril* var. *subsessilis–*BRAZIL, Amazonas**, Manaus, *W.A.Rodrigues 7906* (INPA). **Mato Grosso**, Aripuanã, *M.Gomes 576* (INPA). **Pará**, Porto Trombetas, *H.C. de Lima 6808* (RB); *S.M. de Faria 1241* (RB).

***Hymenaea courvaril* var. *villosa–*BRAZIL, Paraíba**, Near Paquivera de Netuba, *Y-T.Lee 82* (NY).

## References

[B1] Anderson MJ (2001) A new method for non-parametric multivariate analysis of variance. Austral Ecology 26: 32-46

[B2] Andrés-Sánchez S Rico EHA Santos-Vicente M Martínez-Ortega MM (2009) Combining traditional morphometrics and molecular markers in cryptic taxa: towards an updated integrative taxonomic treatment for *Veronica* subgenus *Pentasepalae* (Plantaginaceae sensu APG II) in the western Mediterranean. Botanical Journal of the Linnean Society 159: 68-87. doi: 10.1111/j.1095-8339.2008.00917.x

[B3] Bennett JR Wood JRI Scotland RW (2008) Uncorrelated variation in widespread species: species delimitation in *Strobilanthes echinata* Nees (Acanthaceae). Botanical Journal of the Linnean Society 156: 131-141. doi: 10.1111/j.1095-8339.2007.00756.x

[B4] Bentham G (1870) Leguminosae II: *Swartzieae* et *Caesalpinieae*. In: Martius CFP (org.), Eichler AW, Urban I. Flora Brasiliensis 15 (2): 234-237

[B5] Bruneau A Forest F Herendeen PS Klitgaard BB Lewis GP (2001) Phylogenetic relationships in the Caesalpinioideae (Leguminosae) as inferred from chloroplast *trnL* intron sequences. Systematic Botany 26: 487-514

[B6] Bruneau A Mercure M Lewis GP Herendeen PS (2008) Phylogenetic patterns and diversification in the caesalpinioid legumes. Botany 86: 697-718. doi: 10.1139/B08-058

[B7] Castello LV Galetto L (2013) How many taxa can be recognized within the complex *Tillandsia capillaris* (Bromeliaceae, Tillandsioideae)? Analysis of the available classifications using a multivariate approach. PhytoKeys 23: 25-39. doi: 10.3897/phytokeys.23.4507 23805053 10.3897/phytokeys.23.4507 PMC3690979

[B8] Ceolin GB Miotto STS (2012) Combining ecological and morphometrical approaches to increase the resolution within the *Galactia neesii* (Leguminosae) complex. Plant Systematics and Evolution 298: 645-652. doi: 10.1007/s00606-011-0573-5

[B9] Dayrat B (2005) Towards integrative taxonomy. Biological Journal of the Linnean Society 85: 407-415. doi: 10.1111/j.1095-8312.2005.00503.x

[B10] De Queiroz K (2005) Different species problems and their resolution. BioEssays 27: 1263-1269. doi: 10.1002/bies.20325 16299765 10.1002/bies.20325

[B11] De Queiroz K (2007) Species concepts and species delimitation. Systematic Biology 56: 879-886. doi: 10.1080/10635150701701083 18027281 10.1080/10635150701701083

[B12] Ducke A (1935) As especies brasileiras de jatahy, jutahy ou jatobá (genero *Hymenaea* L., leguminosas cesalpiniaceas). Annaes da Academia Brasileira de Sciencias 7 (3): 203-212

[B13] Egler W (1963) Adolpho Ducke–Traços biográficos, viagens e trabalhos. Boletim do Museu Paraense Emílio Goeldi 18: 1-129

[B14] Estrella M Aedo C Velayos M (2009) A morphometric analysis of *Daniellia* (Fabaceae–Caesalpinioideae). Botanical Journal of the Linnean Society 159: 268-279. doi: 10.1111/j.1095-8339.2008.00894.x

[B15] Fougère-Danezan M Maumont S Bruneau A (2007) Relationships among resin-producing Detarieae s.l. (Leguminosae) as inferred by molecular data. Systematic. Botany 32: 748-761. doi: 10.1600/036364407783390755

[B16] Hammer Ø Harper DAT Ryan PD (2001) PAST: Paleontological Statistics Software Package for Education and Data Analysis. Palaeontologia Electronica 4(1): 9pp.

[B17] Hamilton CW Reichard SH (1992) Current practice in the use of subspecies, variety and forma in the classification of wild plants. Taxon 41: 485-498. doi: 10.2307/1222819

[B18] Henderson A (2006) Traditional morphometrics in plant systematics and its role in palm systematics. Botanical Journal of the Linnean Society 151: 103-111. doi: 10.1111/j.1095-8339.2006.00526.x

[B19] Hijmans RJ Guarino L Jarvis A O’Brien R Mathur P Bussink C Cruz M Barrantes I Rojas E (2005) DIVA-GIS 7.1.7. http://www.diva-gis.org [accessed 01 jun 2013]

[B20] Index Herbariorum (2013) Index Herbariorum Parte II: The Herbaria of the World. Available at: http://sweetgum.nybg.org/ih/ (accessed: 30 jun. 2013).

[B21] Lee Y-T Langenheim JH (1974) Additional new taxa and combinations in *Hymenaea* (Leguminosae, Caesalpinioideae). Journal of the Arnold Arboretum 55 (3): 441-452

[B22] Lee Y-T Langenheim JH (1975) Systematics of the genus *Hymenaea L*. (Leguminosae, Caesalpinioideae, Detarieae). University of California Publications in Botany 69: 109.

[B23] Lima HC de (2013)*Hymenaea*, Lista de Espécies da Flora do Brasil. Jardim Botânico do Rio de Janeiro. http://floradobrasil.jbrj.gov.br/jabot/floradobrasil/FB22971 [accessed 30 July 2013]

[B24] Mackinder B (2005) Detarieae. In: Legumes of the world. In: Lewis G, Schrire B, MacKinder B, Lock M (Eds) The Royal Botanical Gardens, Kew, UK 69–71.

[B25] McNeill J Barrie CFR Buck WR Demoulin V Greuter W Hawksworth DL Herendeen PS Knapp S Marhold K Prado J Prud’Homme van Reine WF Smith GF Wiersema JH (2011) International Code of Nomenclature for algae, fungi and plants (Melborne Code). http://ibot.sav.sk/icbn/main.html [accessed 21 Nov 2013]

[B26] Newmaster SG RagupathyS (2009) Testing plant barcoding in a sister species complex of pantropical *Acacia* (Mimosoideae, Fabaceae). Molecular Ecology Resources 9: 172-180. doi: 10.1111/j.1755-0998.2009.02642.x 21564976 10.1111/j.1755-0998.2009.02642.x

[B27] Nosil P (2008) Speciation with gene flow could be common. Molecular Ecology 17: 2103-2106. doi: 10.1111/j.1365-294X.2008.03715.x 18410295 10.1111/j.1365-294X.2008.03715.x

[B28] Pedersen HÆ (2010) Species delimitation and recognition in the *Brachycorythis helferi* complex (Orchidaceae) resolved by multivariate morphometric analysis. Botanical Journal of the Linnean Society 162: 64-76. doi: 10.1111/j.1095-8339.2009.01015.x

[B29] Pennington TD (1969) Materials for a monograph of the Meliaceae I. A revision of the genus *Vavaea*. Blumea 17: 351-366

[B30] Pestana LTC (2010) Estudo taxonômico de *Hymenea* L.: complexo *H. courbaril*, *H. martiana* e *H. stigonocarpa* (Fabaceae: Caesalpinioidea: Detarieae). Masters thesis, Universidade Federal de Mato Grosso do Sul, Campo Grande, 45.

[B31] Queiroz LP (2009) Leguminosas da Caatinga. Universidade Estadual de Feira de Santana, Feira de Santana, 443p.

[B32] Rahman MDZ Rahman MO (2012) Morphometric analysis of *Desmodium* Desv. in Bangladesh. Bangladesh Journal of Botany 41 (2): 143-148

[B33] Ramos ACS Lemos-Filho JP Lovato MB (2009) Phylogeographical structure of the neotropical forest tree *Hymenaea courbaril* (Leguminosae: Caesalpinioideae) and its relationship with the vicariant *Hymenaea stigonocarpa* from Cerrado. Journal of Heredity 100 (2): 206-216. doi: 10.1093/jhered/esn092 18974401 10.1093/jhered/esn092

[B34] Ramos ACS Lemos-Filho JP Ribeiro RA Santos FR Lovato MB (2007) Phylogeography of the tree *Hymenaea stigonocarpa* (Fabaceae: Caesalpinioideae) and the influence of Quaternary climate changes in the Brazilian Cerrado. Annals of Botany 100: 1219-1228. doi: 10.1093/aob/mcm221 17881340 10.1093/aob/mcm221 PMC2759258

[B35] Rizzini CT (1978) Plantas do Brasil: árvores e madeiras úteis do Brasil - manual de dendrologia brasileira. Edgar Blucher: São Paulo, 296p.

[B36] Rocha DMS (1988) Estudo filogenético de *Hymenaea* L. baseado em proteínas de semente. Masters thesis, Universidade Estadual de Campinas, Campinas, 213p.

[B37] Schlick-Steiner BC Seifert B Stauffer C Christian E Crozier RH Steiner FM (2007) Without morphology, cryptic species stay in taxonomic crypsis following discovery. Trends in Ecology Evolution 22: 391-392. doi: 10.1016/j.tree.2007.05.004 17573150 10.1016/j.tree.2007.05.004

[B38] Scrivanti LR Norrmann GA Anton AM (2013) Delimiting species boundaries within the *Bothriochloa saccharoides* complex (Poaceae) through morphometric analysis. Phytotaxa 89: 24-42. doi: 10.11646/phytotaxa.89.1.2

[B39] SpeciesLink. Available at http://specieslink.org.br [Accessed 10 Jul 2013]

[B40] Wheeler QD Raven PH Wilson EO (2004) Taxonomy: Impediment or Expedient? Science 303: 285. doi: 10.1126/science.303.5656.285 10.1126/science.303.5656.285 14726557

[B41] Whitmore TC (1976) Natural variation and its taxonomic treatment within tropical tree species as seen in the Far East. In: Burley J Styles BT (Eds). Tropical trees.Variation, breeding and conservation. Academic Press, London, 25-34

